# The combination of *HLA*-B*15:01 and DRB1*15:01 is associated with gemcitabine plus erlotinib-induced interstitial lung disease in patients with advanced pancreatic cancer

**DOI:** 10.1007/s00280-016-3026-6

**Published:** 2016-04-21

**Authors:** Meiko Nishimura, Masanori Toyoda, Kei Takenaka, Yoshinori Imamura, Naoko Chayahara, Naomi Kiyota, Toru Mukohara, Takeshi Kotake, Akihito Tsuji, Kosuke Saito, Yoshiro Saito, Hironobu Minami

**Affiliations:** Division of Medical Oncology/Hematology, Kobe University Graduate School of Medicine, Kobe, Japan; Cancer Center, Kobe University Hospital, Kobe, Japan; Department of Medical Oncology, Kobe City Hospital Organization, Kobe City Medical Center General Hospital, Kobe, Japan; Division of Medicinal Safety Science, National Institute of Health Sciences, Tokyo, Japan; Department of Breast Surgery, Kyoto University Hospital, Kyoto, Japan; Department of Clinical Oncology, Faculty of Medicine, Kagawa University, Kagawa, Japan

**Keywords:** Erlotinib, Gemcitabine, Human leukocyte antigen, Interstitial lung disease, Pancreatic cancer

## Abstract

**Purpose:**

In a phase III study of gemcitabine plus erlotinib for advanced pancreatic cancer conducted in Canada, the incidence of interstitial lung disease (ILD) was 3.5 %. However, the incidence of ILD was reported as high as 8.5 % in a Japanese phase II study. These results suggest the influence of ethnic factors in the association of the use of gemcitabine plus erlotinib with the incidence of ILD. Here, we conducted a prospective study to analyze the relationship between human leukocyte antigen (HLA) alleles and ILD in Japanese patients with advanced pancreatic cancer receiving gemcitabine plus erlotinib.

**Methods:**

Patients were treated with gemcitabine (1000 mg/m^2^; administered by intravenous infusion on days 1, 8, and 15 every 4 weeks) and erlotinib (given orally at 100 mg/day). We compared the frequencies of HLA alleles in patients who did and did not develop ILD.

**Results:**

A total of 57 patients were treated, and 4 patients (7.0 %) developed ILD. The combination of *HLA*-B*15:01 and DRB1*15:01 was observed in 2 of 4 patients (50 %) with ILD and in only 1 of 53 patients without ILD (2 %) resulting in odds ratio of 52.0 (95 % CI 3.2–842.5; *p* = 0.011).

**Conclusion:**

These results suggest that the combination of *HLA*-B*15:01 and DRB1*15:01 is associated with ILD in Japanese patients with advanced pancreatic cancer receiving gemcitabine plus erlotinib.

**Electronic supplementary material:**

The online version of this article (doi:10.1007/s00280-016-3026-6) contains supplementary material, which is available to authorized users.

## Introduction

In a phase III study, gemcitabine plus erlotinib improved overall survival significantly compared with gemcitabine alone in patients with advanced pancreatic cancer [1]. As single agents, gemcitabine and erlotinib cause interstitial lung disease (ILD), which may be life threatening. A higher incidence of ILD was reported in Japanese patients compared to white patients treated with either erlotinib or gemcitabine. In patients with non-small cell lung cancer, erlotinib treatment resulted in ILD in 4.3 % of Japanese patients and 2.7 % of white patients [2, 3]. Similarly, the incidence of ILD in patients treated with gemcitabine for pancreatic cancer was 2.1 % of Japanese patients and 0.4 % of white patients [1, 4]. In a phase III study of combination chemotherapy with gemcitabine and erlotinib for pancreatic cancer conducted in Canada, the incidence of ILD was 3.5 %, while a higher incidence of 8.5 % was reported in a Japanese phase II study of the same chemotherapy [5]. Although the etiology of gemcitabine plus erlotinib-induced ILD is unclear, the higher incidence of ILD in Japanese cancer patients suggests an interethnic difference.

Risk factors for ILD identified in a post-marketing surveillance study of erlotinib plus gemcitabine (POLARIS) in Japanese pancreatic cancer patients were the number of metastatic sites at **>**3 [hazard ratio (HR) 4.2 (95 % CI 2.2–8.2)] and concurrent/previous lung diseases [HR 2.2 (95 % CI 1.1–4.5)] [6]. In a phase IV surveillance study of erlotinib (POLARSTAR) as a single agent in Japanese patients with non-small cell lung cancer, concurrent/previous ILD [HR 3.2 (95 % CI 2.4–4.3)], emphysema or chronic obstructive pulmonary disease [HR 1.9 (95 % CI 1.4–2.4)], lung infection [HR 1.6 (95 % CI 1.1–2.2)], smoking history [HR 2.3 (95 % CI 1.7–3.0)], and period from initial cancer diagnosis to the start of the treatment [<360 days; HR 0.6 (95 % CI 0.5–0.7)] were associated with ILD [2]. For gemcitabine, prior thoracic radiotherapy [HR 26.3 (95 % CI 3.4–202.1)] and pre-existing pulmonary fibrosis [HR 6.5 (95 % CI 1.1–38.1)] were identified as significant risk factors for developing ILD in Japanese patients with non-small cell lung cancer and pancreatic cancer [7].

The factors described above do not explain the higher incidence of ILD in Japanese patients treated with gemcitabine, erlotinib, or their combination compared to that of white patients. However, genetic factors may explain the observed interethnic difference in ILD. A genome-wide linkage study identified the mucin (*MUC*) *5B* gene as associated with familial interstitial pneumonia and idiopathic pulmonary fibrosis (IPF) in a white population [8], while an association between the *MUC4* gene and ILD induced by epidermal growth factor receptor tyrosine kinase inhibitors (EGFR-TKI) or acute exacerbation of IPF was reported in Japanese patients [9]. A few previous studies also reported the association between human leukocyte antigen (HLA) alleles and IPF or drug hypersensitivity [10–16]. However, a relationship between HLA alleles and anticancer drug-induced ILD has not been elucidated. To elucidate genetic backgrounds correlated with a higher incidence of ILD by gemcitabine and erlotinib in Japanese patients, we conducted a prospective study to analyze the association between HLA alleles and ILD in patients with advanced pancreatic cancer receiving gemcitabine plus erlotinib.

## Patients and methods

### Patients

Patients (20–80 years old) with histological or cytological evidence of unresectable locally advanced or metastatic pancreatic cancer were enrolled. Other eligibility criteria included an Eastern Cooperative Oncology Group (ECOG) performance status (PS) of 0–1, adequate hematologic, renal, and hepatic functions, and a life expectancy of at least 2 months. Patients with a concurrent or previous history of ILD, IPF, pneumoconiosis, drug-induced pneumonia, pulmonary emphysema, or chronic obstructive pulmonary disease were excluded. Patients treated with radiation to the chest or lung resection, as well as gemcitabine or EGFR-TKI within 3 months, were also excluded.

### Study design and treatment

Patients were treated with gemcitabine (1000 mg/m^2^ by intravenous infusion for 30 min on days 1, 8, and 15 every 4 weeks) and erlotinib (given orally at 100 mg/day) [1]. The treatment continued until disease progression, unacceptable toxicities, or refusal by patients. This study was approved by the Institutional Review Boards of Kobe University Hospital, Kobe City Medical Center General Hospital, and National Institute of Health Sciences. All patients provided written informed consent.

### Samples and HLA typing

Blood samples were collected from all patients within the 2 weeks before starting the treatment. DNA for HLA typing was extracted from peripheral lymphocytes. HLA-A, B, and DRB1 alleles were determined using the Luminex 200 system (Luminex, Austin, TX, USA) and WAKFlow HLA typing kit (Wakunaga, Hiroshima, Japan). Data were analyzed using WAKFlow typing software (Wakunaga, Hiroshima, Japan) in the HLA Foundation Laboratory (Kyoto, Japan) [17].

### Assessments

Chest X-ray was performed weekly for the first 4 weeks and every 2 weeks thereafter. Chest computed tomography (CT) scan was performed every 4 weeks. Antitumor efficacy was evaluated by CT every 8 weeks based on the Response Evaluation Criteria in Solid Tumors (RECIST), version 1.1. Adverse events were assessed using Common Terminology Criteria for Adverse Events (CTCAE), version 4.0.

### Statistical analysis

Statistical analyses were performed using SPSS software version 21.0. Allele frequencies of HLA in patients with or without ILD were compared using Fisher’s exact test with 2 × 2 tables. *P* values <0.05 were considered statistically significant.

We considered that at least 4 ILD events were necessary for the association study. In a Japanese phase II study of gemcitabine plus erlotinib for pancreatic cancer, the incidence of ILD was 8.5 %; therefore, the original planned sample size was approximately 50 to detect 4 ILD events.

## Results

Between February 2013 and July 2015, 57 patients were enrolled from two institutions: Kobe University Hospital (*n* = 44) and Kobe City Medical Center General Hospital (*n* = 13). Of these patients, 4 patients (7.0 %) developed ILD. Baseline characteristics are shown in Table 1. Median age and smoking history were similar between the two groups.

**Table 1:** Baseline characteristics

Characteristic	ILD (*N* = 4)	Non-ILD (*N* = 53)	Total (*N* = 57)
Age—years			
Median	67	65	66
Range	64–76	25–80	25–80
Age group—no. (%)			
<60 years	0 (0)	9 (17)	9 (16)
≥60 years	4 (100)	44 (83)	48 (84)
Sex—no. (%)			
Female	2 (50)	26 (49)	28 (49)
Male	2 (50)	27 (51)	29 (51)
ECOG PS—no. (%)			
0	2 (50)	8 (15)	10 (18)
1	2 (50)	45 (85)	47 (82)
Smoking history—no. (%)			
Never smoker	2 (50)	25 (47)	27 (47)
Ex-smoker	2 (50)	24 (45)	26 (46)
Current smoker	0 (0)	4 (8)	4 (7)
Extent of disease—no. (%)			
Metastatic	3 (75)	32 (60)	35 (61)
Locally advanced	1 (25)	21 (40)	22 (39)
Site of metastatic disease—no. (%)			
Lung	1 (25)	8 (15)	9 (16)
Liver	3 (75)	21 (40)	23 (40)
Peritoneum	0 (0)	14 (26)	14 (25)
Other	0 (0)	1 (2)	1 (2)
Number of metastatic site—no. (%)			
<3	4 (100)	51 (96)	55 (96)
≥3	0 (0)	2 (4)	2 (4)

*ILD* interstitial lung disease, *ECOG PS* Eastern Cooperative Oncology Group performance status

The median duration of the treatment was 1.7 months (range 1.0–3.3) in patients who developed ILD and 2.7 (0.1–16.0) months in those without ILD. The most common reason for discontinuation was disease progression (45 patients, 78.9 %) evaluated by RECIST criteria. Treatment-related adverse events lead to treatment discontinuation in 7 patients (12.3 %) due to malaise, diarrhea, dysgeusia, and ILD (4 patients, 7.0 %). Treatment was discontinued due to adverse events not related to the treatment in 4 patients (7.0 %). One patient (1.8 %) stopped the treatment due to progressive symptoms of the primary disease.

Median progression-free survival was 2.8 (1.0–3.5) months in patients with ILD and 2.8 (0.1–16.8) months in patients without ILD. Partial responses were achieved in 2 patients (3.5 %), and stable disease (SD) was observed in 27 patients (47.4 %). Best response in four patients who developed ILD was SD in 1 and disease progression in 3 patients.

**Fig. 1:**
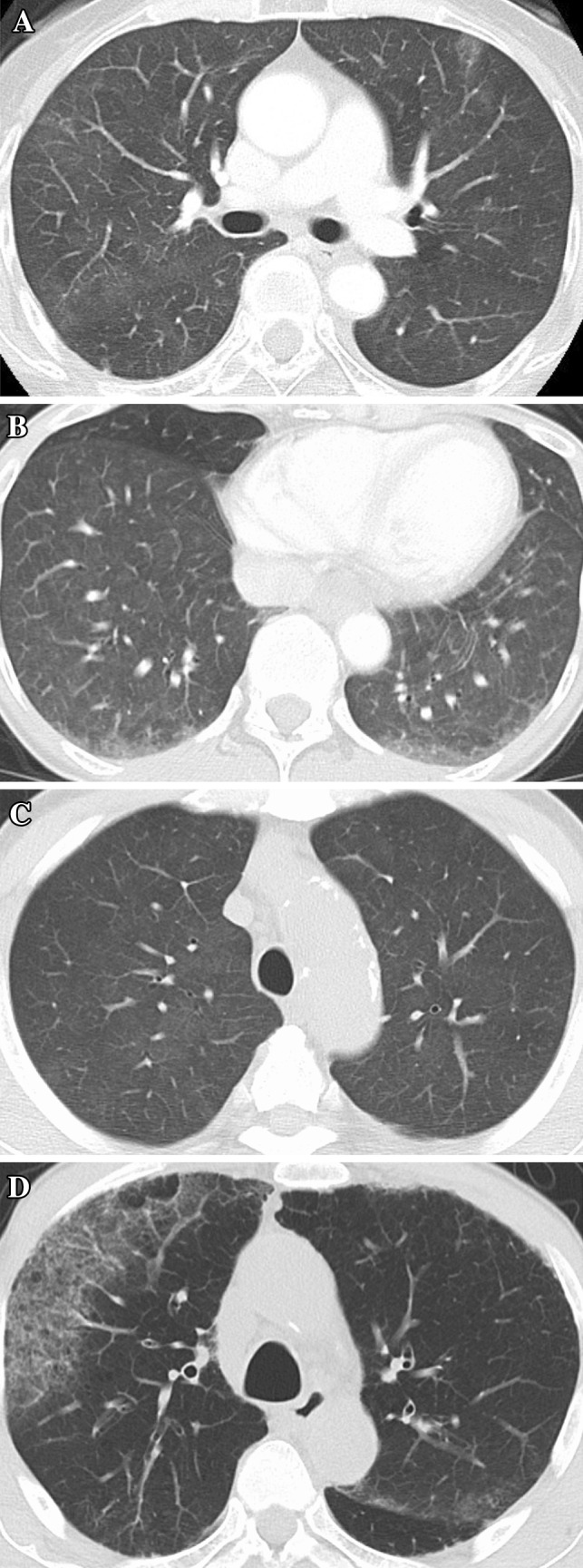
**a** Chest CT scans of a 65-year-old female non-smoker with lung and liver metastases at 8 weeks. **b** Chest CT scans of a 64-year-old female non-smoker with locally advanced cancer at 12 weeks. **c** Chest CT scans of a 76-year-old male ex-smoker with lung metastasis at 4 weeks. **d** Chest CT scans of a 70-year-old male ex-smoker with liver metastasis at 6 weeks

Chest CT scans of 4 patients who developed ILD at the onset of ILD are shown in Fig. 1. ILD developed at 4, 6, 8, and 12 weeks after the start of the treatment in the 4 patients, respectively. ILD was asymptomatic in 1 patient (grade 1), while it was associated with mild symptoms including productive cough, dyspnea, or fever in 3 patients (grade 2). ILD was improved by treatment discontinuation in all patients.

In HLA analysis, 2 of 4 patients who developed ILD harbored *HLA*-B*15:01, B*40:06, DRB1*09:01, and DRB1*15:01, but their association with ILD was not statistically significant. The frequencies of HLA alleles in patients with or without ILD are listed in the Supplementary Table. However, the combination of *HLA*-B*15:01 and DRB1*15:01 was observed in 2 of the 4 patients (50 %) who developed ILD, while only 1 of 53 patients (2 %) who did not develop ILD harbored this combination, resulting in an odds ratio (OR) of 52.0 (95 % CI 3.2–842.5; *p* = 0.01, Table 2).

**Table 2:** The allele frequencies (AF) of a combination of HLA in patients who developed ILD and those without ILD with an OR **>** 2.0

HLA allele	ILD (2*N* = 8)		Without ILD (2*N* = 106)		RR	OR (95 % CI)	*p* value	AF in the study (%)
	Positive (*N*)	AF in ILD (%)	Positive (*N*)	AF in without ILD (%)				
A*11:01/A*31:01	1	25.0	2	3.8	6.0	8.5 (0.6–122.5)	0.20	5.3
DRB1*15:01/DRB1*09:01	1	25.0	1	1.9	9.2	17.3 (0.9–350.3)	0.14	3.5
A*02:10/B*40:06	1	25.0	1	1.9	9.2	17.3 (0.9–350.3)	0.14	3.5
A*11:01/B*15:01	1	25.0	2	3.8	6.0	8.5 (0.6–122.5)	0.20	5.3
A*11:01/B*51:01	1	25.0	1	1.9	9.2	17.3 (0.9–350.3)	0.14	3.5
A*24:02/B*40:06	1	25.0	1	1.9	9.2	17.3 (0.9–350.3)	0.14	3.5
A*26:03/B*15:01	1	25.0	1	1.9	9.2	17.3 (0.9–350.3)	0.14	3.5
A*31:01/B*15:01	1	25.0	1	1.9	9.2	17.3 (0.9–350.3)	0.14	3.5
A*31:01/B*51:01	1	25.0	5	9.4	2.8	3.2 (0.3–36.8)	0.37	10.5
A*33:03/B*44:03	1	25.0	4	7.5	3.5	4.1 (0.3–48.9)	0.32	8.8
A*24:02/DRB1*09:01	1	25.0	6	11.3	2.4	2.6 (0.2–29.3)	0.42	13.2
A*24:02/DRB1*15:01	1	25.0	4	7.5	3.5	4.1 (0.3–48.9)	0.32	8.8
A*26:01/DRB1*09:01	1	25.0	1	1.9	9.2	17.3 (0.9–350.3)	0.13	3.5
A*26:03/DRB1*09:01	1	25.0	2	3.8	6.0	8.5 (0.6–122.5)	0.20	5.3
A*31:01/DRB1*15:01	1	25.0	1	1.9	9.2	17.3 (0.9–350.3)	0.14	3.5
A*33:03/DRB1*04:05	1	25.0	2	3.8	6.0	8.5 (0.6–122.5)	0.20	5.3
A*33:03/DRB1*13:02	1	25.0	1	1.9	9.2	17.3 (0.9–350.3)	0.14	3.5
B*15:01/DRB1*09:01	1	25.0	3	5.7	4.4	5.6 (0.4–70.8)	0.26	7.0
B*15:01/DRB1*15:01	2	50.0	1	2.0	34.0	52.0 (3.2–842.5)	0.01	5.3
B*40:06/DRB1*04:05	1	25.0	2	3.8	6.0	8.5 (0.6–122.5)	0.20	5.3
B*40:06/DRB1*09:01	1	25.0	3	5.7	4.4	5.6 (0.4–70.8)	0.26	7.0

*AF* allele frequencies, *RR* relative risk, *OR* odds ratio, *CI* confidence interval

## Discussion

Our results demonstrate that the combination of *HLA*-B*15:01 and DRB1*15:01 is over-represented significantly in Japanese patients with advanced pancreatic cancer who developed ILD after the treatment with gemcitabine plus erlotinib. Although the number of patients was small, the OR of 52.0 was much higher than the OR (2.2–4.2) of previously reported risk factors for ILD caused by gemcitabine and erlotinib [6].

An over**-**representation of *HLA*-DRB1*15:01 was reported in white patients with IPF not associated with drug therapy [10]. IPF is a clinical form of ILD and the most common type of ILD. Other HLA types (*HLA*-A*3, B*14, B*15, or B*40, and combination of *A2B15*, *A2B40*, *A11B15*, *A24B58*, or *A30B40*) were also associated with IPF in a Han Chinese population [11]. Therefore, drug-induced ILD may be associated with certain types of HLA.

HLA alleles are associated with drug hypersensitivity. Carbamazepine-induced Stevens–Johnson syndrome is associated with *HLA*-B*15:02 in Chinese patients and *HLA*-A*31:01 in Japanese or European patients [12–14]. *HLA*-B*58:01 and *HLA*-B*57:01 are over-represented in allopurinol-induced severe cutaneous reactions [15] and abacavir hypersensitivity [16], respectively. Among anticancer drugs, hepatotoxicity by lapatinib is associated with *HLA*-DQA1*02:01 or DRB1*07:01 [18]. The frequencies of these alleles are 10–15 % in white patients and 0.3–0.8 % in Japanese patients [17, 19].

With regard to drug-induced ILD, *HLA*-A*31:01 and *HLA*-DRB1*15:02 are known to be associated with methotrexate-induced ILD in Japanese patients with rheumatoid arthritis (RA), which induce ILD as a complication [20, 21], and DRB1*15 and *16 were associated with a risk of ILD in Japanese RA patients regardless of methotrexate treatment [22].

These observations suggest that HLA plays an important role in drug-induced ILD, and our findings demonstrate a significant association of certain HLA types with drug-induced ILD. The mechanism of the association between HLA and ILD is still unclear, although an in vitro study demonstrated that a direct interaction between HLA and carbamazepine activates T cells [23]. Drugs or their metabolites may act as haptens and non-covalently bind to the HLA molecule [24].

The incidence of ILD by gemcitabine plus erlotinib for advanced pancreatic cancer was 8.5 % in a Japanese phase II study, 6.2 % in a post-marketing surveillance study, and 7.0 % in our study. We carefully excluded patients with risk factors for ILD from our study. Nonetheless, a significant number of patients developed ILD. These incidences are obviously higher than those reported in white patients. In our prospective study, an association between drug-induced ILD and *HLA*-B*15:01/DRB1*15:01 was observed. Differences in the population frequency of the HLA alleles may explain, at least in part, the interethnic differences in the incidence of ILD induced by gemcitabine and erlotinib.

In conclusion, these results suggest that the combination of *HLA*-B*15:01 and DRB1*15:01 is associated with ILD in Japanese patients with advanced pancreatic cancer receiving gemcitabine plus erlotinib.

## Electronic supplementary material

Below is the link to the electronic supplementary material.

Supplementary material 1 (DOC 103 kb)
