# Supplementary material for: The combination of HLA-B*15:01 and DRB1*15:01 is associated with gemcitabine plus erlotinib-induced interstitial lung disease in patients with advanced pancreatic cancer
Source: Cancer Chemother Pharmacol. 2016 Apr 21;77:1165–70. doi: 10.1007/s00280-016-3026-6 (PMC4882349; doi:10.1007/s00280-016-3026-6)
Supplement: Supplementary file 1 — Supplementary material 1 (DOC 103 kb) [file 280_2016_3026_MOESM1_ESM.doc]

**Supplemental Table** The allele frequencies (AF) of HLA of more than 10% in patients with or without ILD.

| HLA allele | ILD  (2*N* = 8) | | Without ILD  (2*N* = 106) | | RR | OR (95% CI) | *P* value | AF in the study  (%) | AF in Japanese (%) | AF in Caucasian (%) |
| --- | --- | --- | --- | --- | --- | --- | --- | --- | --- | --- |
|  | Positive (*N*) | AF in ILD (%) | Positive (*N*) | AF in without ILD  (%) |  |  |  |  |  |  |
| *A*02:01* | 0 | 0 | 11 | 20.8 | - | - | - | 19.3 | 11.6 | 27.2 |
| *A*02:06* | 0 | 0 | 10 | 18.9 | - | - | - | 17.5 | 9.1 | 0.2 |
| *A*11:01* | 1 | 25.0 | 8 | 15.1 | 1.8 | 1.9 (0.2 - 20.4) | 0.51 | 15.8 | 9.1 | 7.0 |
| *A*24:02* | 1 | 25.0 | 28 | 52.8 | 0.3 | 0.3 (0.3 - 3.1) | 0.35 | 50.8 | 36.5 | 6.6 |
| *A*26:01* | 1 | 25.0 | 6 | 11.3 | 2.4 | 2.6 (0.2 - 29.3) | 0.42 | 12.3 | 7.4 | 4.2 |
| *A*31:01* | 1 | 25.0 | 11 | 20.8 | 1.3 | 1.3 (0.1 - 13.5) | 1.0 | 21.1 | 8.4 | 3.2 |
| *B*15:01* | 2 | 50.0 | 7 | 13.2 | 5.3 | 6.6 (0.8 - 54.5) | 0.11 | 15.8 | 7.6 | 6.0 |
| *B*35:01* | 0 | 0 | 13 | 24.5 | - | - | - | 22.8 | 8.3 | 6.8 |
| *B*40:01* | 0 | 0 | 6 | 11.3 | - | - | - | 10.5 | 5.3 | 6.8 |
| *B*40:06* | 2 | 50.0 | 4 | 7.5 | 8.5 | 12.3 (1.4 - 111.6) | 0.05 | 10.5 | 4.8 | 0.0 |
| *B*51:01* | 1 | 25.0 | 10 | 18.9 | 2.0 | 1.4 (0.1 - 15.3) | 1.0 | 19.3 | 8.9 | 5.7 |
| *B*52:01* | 0 | 0 | 11 | 20.8 | - | - | - | 19.3 | 11.1 | 1.1 |
| *B*54:01* | 0 | 0 | 7 | 13.2 | - | - | - | 12.3 | 7.5 | 0.0 |
| *DRB1*01:01* | 0 | 0 | 6 | 11.3 | - | - | - | 10.5 | 5.8 | 8.7 |
| *DRB1*04:05* | 1 | 25.0 | 18 | 34.0 | 0.7 | 0.7 (0.1 - 6.7) | 1.0 | 33.3 | 13.5 | 0.4 |
| *DRB1*08:02* | 0 | 0 | 6 | 11.3 | - | - | - | 10.5 | 4.2 | 0.0 |
| *DRB1*08:03* | 0 | 0 | 10 | 18.9 | - | - | - | 17.5 | 8.3 | 0.0 |
| *DRB1*09:01* | 2 | 50.0 | 14 | 26.4 | 4.8 | 2.8 (0.4 - 21.7) | 0.31 | 28.1 | 14.3 | 1.0 |
| *DRB1*15:01* | 2 | 50.0 | 5 | 9.4 | 7.1 | 9.6 (1.1 - 83.7) | 0.07 | 12.3 | 7.8 | 13.9 |
| *DRB1*15:02* | 0 | 0 | 12 | 22.6 | - | - | - | 21.1 | 10.7 | 0.8 |

**Abbreviations:** AF, allele frequencies; RR, relative risk; OR, Odds ratio; CI, confidence interval;
